# Multimodal Classification Framework Based on Hypergraph Latent Relation for End-Stage Renal Disease Associated with Mild Cognitive Impairment

**DOI:** 10.3390/bioengineering10080958

**Published:** 2023-08-12

**Authors:** Xidong Fu, Chaofan Song, Rupu Zhang, Haifeng Shi, Zhuqing Jiao

**Affiliations:** 1 School of Computer Science and Artificial Intelligence, Changzhou University, Changzhou 213164, China; 2 Department of Radiology, The Affiliated Changzhou No.2 People’s Hospital of Nanjing Medical University, Changzhou 213003, China

**Keywords:** end-stage renal disease, mild cognitive impairment, hypergraph, latent relation adaptive similarity learning, multimodal classification framework

## Abstract

Combined arterial spin labeling (ASL) and functional magnetic resonance imaging (fMRI) can reveal more comprehensive properties of the spatiotemporal and quantitative properties of brain networks. Imaging markers of end-stage renal disease associated with mild cognitive impairment (ESRDaMCI) will be sought from these properties. The current multimodal classification methods often neglect to collect high-order relationships of brain regions and remove noise from the feature matrix. A multimodal classification framework is proposed to address this issue using hypergraph latent relation (HLR). A brain functional network with hypergraph structural information is constructed by fMRI data. The feature matrix is obtained through graph theory (GT). The cerebral blood flow (CBF) from ASL is selected as the second modal feature matrix. Then, the adaptive similarity matrix is constructed by learning the latent relation between feature matrices. Latent relation adaptive similarity learning (LRAS) is introduced to multi-task feature learning to construct a multimodal feature selection method based on latent relation (LRMFS). The experimental results show that the best classification accuracy (ACC) reaches 88.67%, at least 2.84% better than the state-of-the-art methods. The proposed framework preserves more valuable information between brain regions and reduces noise among feature matrixes. It provides an essential reference value for ESRDaMCI recognition.

## 1. Introduction

End-stage renal disease (ESRD) is an end-stage of chronic kidney disease, often accompanied by serious symptoms such as renal failure and multi-organ dysfunction [1]. In recent years, the global incidence of ESRD has been increasing yearly, which brings a heavy economic burden to society and families and great psychological stress to patients [2]. Studies have shown that 30% to 60% of ESRD patients, especially those receiving hemodialysis treatment, develop mild cognitive impairment (MCI) [3]. If left untreated, MCI may develop into Alzheimer’s disease (AD), affecting ESRD patients’ later treatment [4]. However, the pathophysiological mechanisms of ESRD associated with MCI (ESRDaMCI) are not fully understood, so neuroimaging studies of these patients are important for their treatment.

Currently, neuroimaging techniques have matured, allowing researchers to acquire various types of medical imaging data easily. Numerous studies have demonstrated that a more comprehensive understanding of disease mechanisms can be achieved by examining multimodal data from participants [5,6] in contrast to the information in unimodal data. For example, Jiao et al. [7] combined positron emission tomography (PET) and structural magnetic resonance imaging (sMRI) data for feature selection. Li et al. [8] integrated cerebral blood flow (CBF) from arterial spin labeling (ASL) and blood oxygen level-dependent (BOLD) of functional magnetic resonance imaging (fMRI) to replace unimodal data for the classification of mild cognitive impairment (MCI). Jann et al. [9] proposed a connectivity framework between CBF-ASL and BOLD-fMRI, which revealed more comprehensive spatiotemporal and quantitative properties of brain networks through the combination of fMRI and ASL. The better utilization of multimodal data is a hot topic in current studies.

The most crucial aspect in classifying related diseases, such as MCI or ESRDaMCI, is selecting disease-relevant features from multimodal data to improve classification performance. In machine learning, this objective can be achieved by manipulating the properties of the multimodal feature matrix, changing the feature assignment weights, and optimizing feature selection algorithms. On the one hand, Liu et al. [10] constructed the feature matrix with sparse regularization, thereby reducing the impact of redundant features. Rehman et al. [11] improved model productivity by means of feature assignment weights. On the other hand, among numerous feature selection methods, embedded methods are currently the most widely applied [12]. The multi-task learning is applied for small datasets related to medical diseases because it better reveals shared features among different tasks and facilitates data sharing between them, exhibiting good generalization capability [13]. In addition, the feature assignment right is more likely to ignore the correlation between features than multi-task feature selection, which is not suitable for the classification of multimodal data, and it does not reduce the feature dimensionality, which can easily affect the classification results. The multi-task feature selection method is often enhanced by introducing different regularization terms [14]. For instance, Jie et al. [15] proposed a learning method that combines manifold regularization and multi-task feature selection. They embedded a manifold regularization term into the feature selection process by predefined a similarity matrix to realize data sharing. Shao et al. [16] constructed hypergraph regularization terms for each modality within the feature matrix and incorporated them into multi-task learning to reflect high-order relations among subjects. In recent research, Shi et al. [17] introduced adaptive similarity learning, resulting in more accurate similarity matrices. Song et al. [18] introduced topological manifold terms to construct similarity matrices with topological relations.

Nevertheless, the aforementioned methods only construct similarity matrices based on the original feature matrix without considering the presence of noise and outliers in real-world data. They also overlook the incorporation of high-order prior information from brain regions when constructing the multimodal feature matrix. Just changing the attributes of the feature matrix, or optimizing the feature selection algorithm, does not improve the classification performance better. Improving the classification performance, and finding the discriminative brain regions of ESRDaMCI, can provide a scientific basis for medical clinical diagnosis and identification of ESRDaMCI and reduce the time and economic cost. In view of this, we propose a multimodal classification framework based on hypergraph latent relation (HLR) for classifying ESRDaMCI and normal subjects. Firstly, a brain functional network is constructed based on the method of hypergraph manifold, and the feature matrix of fMRI is extracted by graph theory (GT) [19]. Secondly, CBF from ASL is selected as the feature matrix for the second modality. Then, latent relation adaptive similarity learning (LRAS) is embedded into the multi-task feature selection, constructing a multimodal feature selection method based on latent relation (LRMFS). Finally, the selected features from the proposed framework are linearly fused into a multicore support vector machine (MKSVM) for classification. In this study, the hypergraph feature matrix retains high-order features from the brain function network with hypergraph structural information. It focuses on the relational information of multiple brain regions rather than pairwise brain region relationships. LRMFS is applied to uncover the latent relationships within the feature matrix, resulting in the construction of a robust similarity matrix and the selection of well-represented features. These two components constitute the HLR-based multimodal classification framework. Moreover, the framework can also identify discriminative brain regions affected by ESRDaMCI, providing valuable insights for identifying and diagnosing MCI-related diseases.

## 2. Data and Methods

### 2.1. HLR-Based Multimodal Classification Framework

**Figure 1:**
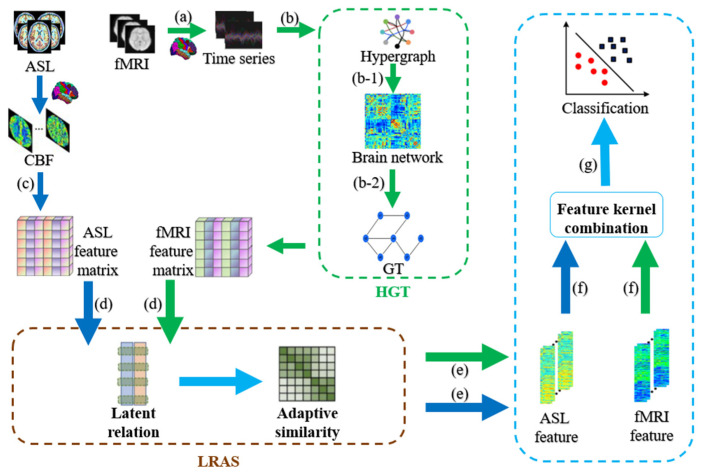
Flowchart of HLR-based multimodal classification framework. Blue arrows represent ASL modality and green arrows represent fMRI modality.

Figure 1 shows the HLR-based multimodal classification framework for ESRDaMCI. The specific steps are as follows. (a) Preprocessing the fMRI data to obtain the time series of all brain regions of the Automated Anatomical Labeling (AAL) template; (b) Constructing the hypergraph feature matrix of fMRI by the hypergraph graph theory (HGT); (b-1) Transforming the Laplacian matrix obtained from hypergraph construction into a hypergraph manifold regularization term and a sparse regularization term to derive a new brain functional network; (b-2) Obtaining the feature matrix of all subjects by graph theory; (c) Extracting CBF values from various brain regions in ASL data with the AAL template to construct a feature matrix; (d) Inputting the feature matrix of two modalities into the multimodal feature selection model proposed by the framework; (e) Outputting well-selected feature vectors from both modalities; (f) Fusing the feature vectors to generate a new feature matrix; (g) Dividing the new feature matrix into training and testing sets, and performing classification on normal subjects and ESRDaMCI patients via MKSVM.

### 2.2. Data Preprocessing

We employ the same original dataset as Song et al. [18]. Forty-four patients with ESRDaMCI from the Affiliated Changzhou No.2 People’s Hospital of Nanjing Medical University were selected as the ESRD group. They include 24 males and 20 females, aged 49.25 ± 11.15 years. Meanwhile, 44 healthy volunteers who underwent a physical examination at the same hospital and matched the age and education level of the subjects in the ESRD group were selected as the Normal Controls (NC) group. They include 13 males and 31 females, aged 46.25 ± 11.39 years.

This study has been approved by the Ethics Committee of this hospital and conformed to the 2013 revision of the Declaration of Helsinki (www.wma.net/en/30publications/10policies/b3/index.html), accessed on 5 May 2023. All subjects signed written informed consent and volunteered to participate in this study. The Montreal Cognitive Assessment (MoCA) assessed their cognitive functions. This assessment tool is primarily used for screening and evaluating cognitive impairments, including attention and concentration, executive functions, memory, language, visuospatial skills, abstract thinking, calculation, and orientation. It is a convenient and effective tool with a total score of 30 points. Scores with 26 or more as normal, 18–26 as mild cognitive impairment, 10–17 as moderate cognitive impairment, and less than 10 as severe cognitive impairment [20]. The ESRD group has an average score of 23.87 ± 4.51 points. Specific participant information can be found in Table 1.

All subjects are scanned by a GE Discovery MR 750W 3.0T scanner and are placed on the magnetic resonance imaging (MRI) equipment. Their heads are immobilized with rubber cork inside the MRI coil during the scans to avoid artifactual images caused by head movements. T1-weighted brain structure images are obtained by adopting the 3D brain volume imaging (3D-BRAVO) sequence. The specific machine parameters are repetition time (TR) = 7.5 ms, echo time (TE) = 2.5 ms, reversal time = 450 ms, flip angle (FA) = 15°, layer interval = 1 mm, the field of view (FOV) = 240 mm × 240 mm, layer thickness = 1 mm, the number of scanning layers = 154, and the scanning time = 3 min 51 s. fMRI is obtained from the gradient-echo plane echo imaging (GRE-EPI) sequence. The machine parameters are TR = 2000 ms, TE = 40 ms, layer thickness = 4 mm, FA = 90°, FOV = 240 mm × 240 mm, matrix size = 64 × 64, and the scanning time = 8 min. ASL is obtained from the 3D pseudo-continuous arterial spin labeling (3D-pcASL) sequence. The machine parameters are TR = 5335 ms, TE = 10.7 ms, layer thickness = 4 mm, post-labeling delay (PLD) = 2525 ms, FOV = 240 mm × 240 mm, layer thickness = 4 mm, the number of scanning layers = 36, and the scanning time = 3 min 44 s.

The raw fMRI data are preprocessed in the Statistical Parametric Mapping (SPM12) and the Data Processing Assistant for Resting-State fMRI (DPARSF) on the Matlab 2021a platform [21]. The preprocessing involves the following steps. (a) Data format conversion: The original sample data in DICOM format are converted to 4D NIFTI format files. (b) Slice timing correction: The first ten time points are discarded because it takes time for the examination instrument and the subject to enter a steady state. (c) Image registration: Head motion correction is performed, and the fMRI images are registered to the Montreal Neurological Institute (MNI) standard brain space by an EPI template (Bounding box: [−90, −126, −72; 90, 90, 108], Voxel Size: [3 3 3]). (d) Spatial smoothing: The normalized fMRI images are spatially smoothed with a Gaussian kernel (FWHM: [5 5 5]). (e) Bandpass filtering and linear detrending. (f) Time series extraction: It adopts the AAL template to extract the time series of 90 brain regions [22].

The raw ASL data are preprocessed in the SPM12 and the Resting-State Functional MRI Data Analysis Toolkit (REST 1.8) toolbox on the Matlab 2021a platform [23]. The preprocessing involves the following steps. (a) Data format conversion: The original sample data in DICOM format are converted to 4D NIFTI format files. (b) Image registration: It performs the registration taking the CBF image as the reference image and the T1 image as the source image. (c) Image segmentation: The registered T1 structural image is segmented. (d) Normalization: It normalizes the CBF image in the MNI standard brain space (Bounding box: [−90, −126, −72; 90, 90, 108], Voxel Size: [3 3 3]). (e) Spatial smoothing: The normalized wCBF image is spatially smoothed with a Gaussian kernel (FWHM: [5 5 5]). (f) CBF value extraction: The REST toolbox extracts feature from the smoothed CBF image. It sets the CBF of 90 brain regions as the features of ASL.

### 2.3. Hypergraph Feature Matrix

A basic brain functional network is constructed according to *Pearson* correlation coefficients. Then, the Laplacian matrix is obtained by constructing the hypergraph, which is transformed into the hypergraph manifold regularization term. It is introduced into the basic brain functional network with the sparse regularization term [24] to obtain a brain functional network with sparse and hypergraph manifold regularization (SHMR). The objective function of SHMR is:

(1) $$\underset{P_{W}}{\min}∥P_{W}-Q_{B}{}^{T}Q_{B}{∥}_{F}^{2}+\lambda \mathrm{tr}\left(P_{W}{}^{T}L^{h}P_{W}\right)+\gamma ∥P_{W}{∥}_{\;1}$$

where $Q_{B}$ denotes the time series matrix and $P_{W}$ denotes the coefficient matrix of the brain function network. $L^{h}$ is the normalized Laplacian matrix of the hypergraph, λ and γ denote the hypergraph manifold regularization term parameter and sparse regularization term parameter, respectively. λ is set as $2^{-3}$, and γ is set as $2^{-4}$ according to [24].

The brain topological structure is disrupted after the onset of cognitive impairment, leading to decreased transmission efficiency of individual nodes [25]. Thus, we select nodal efficiency as the first modal feature. For each participant’s SHMR-based brain functional network, binary processing is performed by the matrix sparsity as the threshold. The sparsity is set from 0.01 to 0.35 with a step size of 0.01 [26]. The area under the curve (AUC) of nodal efficiency is computed for all participants within the range of sparsity thresholds, resulting in the hypergraph feature matrix. Additionally, we adopt the Z-score normalization method to standardize and normalize the hypergraph feature matrix. The Z-score normalization method can eliminate differences between the CBF features and hypergraph features, facilitating feature weight learning.

### 2.4. Adaptive Similarity Learning

In the case of single-modal data, we assume a feature matrix $X=\left[x_{1},x_{2},\cdots ,x_{n}\right]\in R^{d\times n}$ and a label matrix $y=\left[y_{1},y_{2},\cdots ,y_{n}\right]\in R^{1\times n}$ corresponding to the data, where *d* represents the feature dimension and *n* represents the number of features. The similarity vector ***s*** is constructed by calculating the Euclidean distance between different pairs of feature dimensions. Additionally, it is assumed that the smaller the distance $∥x_{i}-x_{j}{∥}_{\;2}^{2}$ between feature vectors $x_{i}$ and $x_{j}$, the larger the similarity $s_{ij}$. If feature vectors $x_{i}$ and $x_{j}$ belong to different classes, then $s_{ij}=0$. The objective function is defined as follows:

(2) $$\left\{\begin{array}{l} \underset{\forall i,s_{i}}{\min}\sum_{i=1}^{n}\sum_{j=1}^{n}\left(∥x_{i}-x_{j}{∥}_{\;2}^{2}s_{ij}+\alpha s_{ij}^{2}\right)\\ s.t.\;s_{i}^{T}=1,\;0\leq s_{ij}\leq 1.\\ s_{ij}=0,\;\mathrm{if}\;y_{i}\neq y_{j}. \end{array}\right.$$

where $s_{i}\in R^{n}$ represents the similarity vector between $x_{i}$ and others and $s_{ij}$ represents the *j*-th element of $s_{i}$. The objective function of adaptive similarity learning can calculate the similarity matrix for each subject, taking into account both cases: when a feature vector has one or more nearest neighbors with the same similarity and when it does not. Generally, we treat the latter case as a regularization term to avoid trivial solutions from the former case.

Once the objective function for the unimodal similarity vector is determined, it can extend the adaptive similarity learning to multimodal learning. We define the number of modalities as *m*, and $X^{m}=\left[x_{1}^{m},x_{2}^{m},\cdots ,x_{n}^{m}\right]$ represents the feature matrix of the *m*-th modality. For the multimodal data, we solve the following problem to obtain the similarity matrix ***S***:

(3) $$\left\{\begin{array}{l} \underset{S}{\min}\sum_{i=1}^{n}\sum_{j=1}^{n}\left(∥x_{i}{}^{m}-x_{j}{}^{m}{∥}_{\;2}^{2}s_{ij}+\alpha s_{ij}^{2}\right)\\ s.t.\;s_{i}^{T}=1,\;0\leq s_{ij}\leq 1.\;\\ s_{ij}=0,\;\mathrm{if}\;y_{i}\neq y_{j}. \end{array}\right.$$

### 2.5. Multimodal Feature Selection Based on Adaptive Similarity Learning

Multi-task learning involves simultaneously learning multiple related tasks and improving learning efficiency by leveraging the shared data among the tasks [12]. The adaptive similarity-based multimodal feature selection (ASMFS) method is proposed by combining adaptive learning and multi-task feature learning, with the objective function defined as follows:

(4) $$\left\{\begin{array}{l} \;\underset{W,S}{\min}\sum_{m}^{M}\sum_{i}^{N}∥y_{i}-w_{m}^{T}x_{i}^{m}{∥}_{2}^{2}+\mu ∥W{∥}_{2,1}\\ +\beta \sum_{i}^{N}\sum_{k\in \left\{k\left|y_{i}=y_{k}\right.\right\}}^{}\left(\sum_{m}^{M}\left(∥w_{m}^{T}x_{i}{}^{m}-w_{m}^{T}x_{k}{}^{m}{∥}_{\;2}^{2}s_{ik}+\alpha s_{ik}^{2}\right)\right)\\ s.t.\;\sum_{k}^{n}s_{ik}=1\;,0\leq s_{ik}\leq 1. \end{array}\right.$$

where $W=\left[w_{1}{}^{1},w_{2}{}^{2},\cdots w_{n}{}^{m}\right]\in R^{d\times m}$ is the feature weight matrix, and $w^{m}$ represents the weight vector of the *m*-th modality. $∥W{∥}_{2,1}$ represents the $l_{2,1}$ norm of the matrix ***W***, denoted as $∥W{∥}_{2,1}=\sum_{i=1}^{d}∥w^{i}{∥}_{2}$, which allows joint feature selection by combining the weights of the same features across different modalities. μ,α,β are regularization parameters that balance the relative weights of the terms in the equation.

### 2.6. Multimodal Feature Selection Based on Latent Relation

During adaptive similarity learning, real data often contain noise and outliers, which can affect the accuracy of the similarity matrix. Therefore, it is necessary to learn the latent relation within the original data, allowing the adaptive similarity learning process to resist noise and filter out anomalies. Currently, semi-negative matrix factorization (SNMF) can extract the latent relation in the original data due to its intuitive interpretation based on parts [27].

We define $U=\left[U_{}{}^{1},U_{}{}^{2},\cdots U_{}{}^{m}\right]$ as the base matrix for modality, which $U^{m}$ represents the base matrix for the *m*-th modality. ***V*** is the complementary coefficient matrix for all modalities. $\eta =\left[\eta^{1},\eta^{2},\cdots ,\eta^{m}\right]$ is the coefficient matrix of latent feature vectors for different modalities, which is calculated adopting the inverse distance weighted strategy. The objective function for learning the latent relation is expressed as follows:

(5) $$\left\{\begin{array}{l} \underset{\left\{U^{m}\right\},V}{\min}\sum_{m=1}^{m}\eta^{m}∥X^{m}-U^{m}V^{T}{∥}_{2,1}\\ s.t.\;\eta^{m}=\frac{1}{2\sqrt{∥X^{m}-U^{m}V^{T}{∥}_{2,1}}},V\geq 0,V^{T}V=I. \end{array}\right.$$

The coefficient matrix ***V*** in Equation (5) can effectively capture the intrinsic features of the original data. Its orthogonality constraint $V^{T}V=I$ helps reduce the influence of outliers and noise [28]. ***V*** can be considered a robust representation of the original data ***X***, and it can substitute for ***X*** in the adaptive similarity learning process. The objective function of the LRMFS is:

(6) $$\left\{\begin{array}{l} \;\underset{W,S\;,U^{m}\;,V}{\min}\sum_{m}^{M}\sum_{i}^{N}∥y_{i}-w_{m}^{T}x_{i}^{m}{∥}_{2}^{2}+\mu ∥W{∥}_{2,1}\\ +\beta \left[\sum_{i}^{N}\sum_{k\in \left\{k\left|y_{i}=y_{k}\right.\right\}}^{}\left(\sum_{m}^{M}\left(∥w_{m}^{T}v_{i}{}^{m}-w_{m}^{T}v_{k}{}^{m}{∥}_{\;2}^{2}s_{ik}+\alpha s_{ik}^{2}\right)\right)+\sum_{m=1}^{m}\eta^{m}∥X^{m}-U^{m}V^{T}{∥}_{2,1}\right]\\ s.t.\sum_{k}^{n}s_{ik}=1\;,0\leq s_{ik}\leq 1.\;V\geq 0,\;V^{T}V=I,rank\left(L_{s}\right)=n-c. \end{array}\right.$$

where *c* is the number of categories and $rank\left(L_{s}\right)$ is the Laplace rank constraint.

This objective function involves constraints such as orthogonality, non-negativity, and $l_{2,1}$ norm. The iterative update algorithm efficiently addresses the objective function [29]. Its constraints are separated by introducing auxiliary variables $E^{\left(m\right)}=X^{m}-U^{m}V^{T}$ and Z=V, and the equivalence is maintained during the update process. Moreover, balancing parameters ζ and $\Lambda^{m}\in R^{d_{m}\times n}$ are introduced, with $\Lambda^{m}\in R^{d_{m}\times n}$ serving as the Lagrange multiplier matrix for the difference between the target variable and auxiliary variable. Each variable is optimized through iterative updates.

(a) Update $U^{\left(m\right)}$:

Update under the constraint of $V^{T}V=I$, the optimization equation is shown below:

(7) $$U^{\left(m\right)}=\left(X^{\left(m\right)}-E^{\left(m\right)}+\frac{1}{\zeta }\Lambda^{\left(m\right)}\right)V$$

(b) Update V:

With the other variables kept fixed, ***V*** is updated, and the optimization equation is changed to:

(8) $$\underset{V^{T}V=I}{\min}∥V-Q{∥}_{F}^{2}$$

where ***Q*** is obtained by Equation (9):

(9) $$Q=\frac{\sum_{m=1}^{M}{\left(X^{\left(m\right)}-E^{\left(m\right)}+\frac{1}{\zeta }\Lambda^{\left(m\right)}\right)}^{T}U^{\left(m\right)}+Z-\frac{1}{\zeta }\Lambda }{m+1}-\frac{2\beta }{\zeta }L_{s}Z$$

The solution of ***V*** is given by

(10) $$V=OH^{T}$$

where ***O*** and ***H*** are the left and right singular values of SVD [30] of ***Q***

(c) Update $E^{\left(m\right)}$:

Ensuring that the other variables are fixed, the optimization formula for $E^{\left(m\right)}$ is

(11) $$\underset{E^{\left(m\right)}}{\min}\frac{\eta^{m}}{\zeta }∥E^{\left(m\right)}{∥}_{2,1}+\frac{1}{2}∥E^{\left(m\right)}-P^{\left(m\right)}{∥}_{F}^{2}$$

where $P^{\left(m\right)}=X^{m}-U^{m}V^{T}+\frac{1}{\zeta }\Lambda^{\left(m\right)}$.

(d) Update ***W***:

Inspired by [31], the elements of Equation (6) related to ***W*** are weighted and iterated. When the element $w_{i,:}$ in row ***W*** is not zero, define $d_{ii}=\frac{1}{2}∥w_{i,:}{∥}_{2}^{-1}$, and obtain the derivative with respect to $∥W{∥}_{2,1}$:

(12) $$\frac{\partial ∥W{∥}_{2,1}}{\partial w_{ij}}=2d_{ii}w_{ij}$$

Defining ***D*** as the diagonal matrix of the diagonal elements $d_{ii}$ after obtaining Equation (12), we get the indefinite integral of Equation (12). Finally, the derivative formula for the part of Equation (6) containing ***W*** is obtained as follows

(13) $$\underset{W}{\min}L\left(W\right)=\sum_{m=1}^{M}\sum_{i}^{n}∥y_{i}-w_{m}^{T}x_{i}^{m}{∥}_{2}^{2}+\mu \mathrm{Tr}\left(W^{T}DW\right)+\beta \sum_{i}^{n}\sum_{k\in \left\{k\left|y_{i}=y_{k}\right.\right\}}^{}\left(\sum_{m}^{M}∥w_{m}^{T}v_{i}{}^{m}-w_{m}^{T}v_{k}{}^{m}{∥}_{\;2}^{2}s_{ik}\right)$$

(e) Update ***S***:

In the same way as updating ***W***, Equation (6) containing ***S*** is extracted and then $d_{ik}=\sum_{m}^{M}∥w_{m}^{T}v_{i}{}^{m}-w_{m}^{T}v_{k}{}^{m}{∥}_{\;2}^{2}$ is defined. The new objective function is finally obtained as follows:

(14) $$\left\{\begin{array}{l} \underset{s_{i}}{\min}s_{i}+\frac{1}{2\alpha }d_{i2}^{2}\\ s.t.\;\sum {}_{k=1}^{n}s_{ik}=1,\;0\leq s_{ik}\leq 1. \end{array}\right.$$

Since the above objective function is a convex function, it can be solved by the Karush-Kuhn-Tucker (KKT) conditions with the Lagrange multiplier method [32]. The final optimal solution obtained as follows:

(15) $$s_{ik}^{*}={\left(-\frac{d_{ik}}{2\alpha_{i}}+\sigma \right)}_{+}=\max\left(-\frac{d_{ik}}{2\alpha_{i}}+\sigma ,0\right)$$

where σ>0 is the Lagrange multiplier. The iterative update process of LRMFS is shown in Algorithm 1.

**Algorithm 1** Objective function optimization algorithmInput:   $X^{m}$//The feature matrix of the *m*-th modality;      $y^{m}$//The label corresponding to the *m*-th modality subjects;      *K*//The adaptive similarity neighbors;      μ//The group sparsity regularization parameter;      *β*//The regularization parameter for adaptive similarity learning.Output: W//The weight matrix of features.Initialize  ***S***//Constructed by Equation (4);While not converges Fix other variables Update ***U*** by Equation (7) with the constraint $V^{T}V=I$ Then Fix other variables     Compute SVD of ***Q*** Update ***V*** by Equation (10) Then Fix other variables     Compute ***P*** Update ***E*** by Equation (11)      Then Fix other variables Define ***D*** Calculated derivative Update ***W*** by Equation (13)      Then Fix other variables KKT conditions Update ***S*** by Equation (15) End while

## 3. Experiment and Analysis

We select MKSVM as the classifier for data classification [33]. MKSVM performs a linear fusion of kernel functions based on a support vector machine (SVM). It has excellent generalization ability and is particularly suitable for small-sample situations like medical data classification.

The ten-fold cross-validation [34], which allows us to fully use the experimental data, is adopted to evaluate the classification performance of each method. Accuracy (ACC), AUC, specificity (SPE), and sensitivity (SEN) are selected as evaluation metrics for classification performance [8]. ACC represents the proportion of correctly classified samples, AUC describes the size of the area under the curve, and SPE and SEN represent the accuracy of classifying negative and positive samples, respectively. It conducted experiments to investigate the impact of different parameters on its classification performance and identified the optimal parameters after determining the evaluation metrics.

### 3.1. Parameters Selection

**Figure 2:**
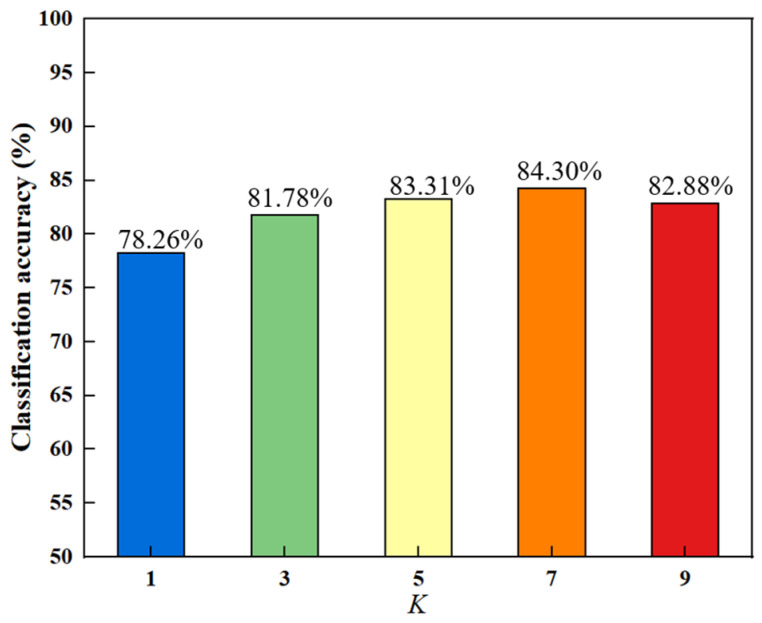
Classification accuracy of different nearest neighbors.

There are a total of one parameter *K* for the number of neighbors, and two regularization parameters μ and *β*. Experimentally, the nearest neighbor number *K* is set to take the values of 1, 3, 5, 7, and 9. The two regularization parameters μ and *β* take the values of {0,5,10,15,20} and {0.1,5,20,60,100}, respectively [17]. We selected an appropriate value for *K* and obtained the results shown in Figure 2 by fixing the values of μ and *β*. Different colors are chosen for the bar chart to make the results more intuitive and beautiful. In further, all experiments are performed by a ten-fold cross-validation method.

In Figure 2, the trend of the bar chart indicates that the overall classification accuracy increases initially and then decreases with the increase of *K*. The best accuracy is obtained when *K* equals 7. The reason for this increasing and then decreasing trend may be that as the number of nearest neighbors increases, the local manifold structure of the data becomes clearer, which helps in selecting discriminative features and improving the accuracy. The manifold structure starts to become unstable after reaching the peak, leading to a decline in accuracy.

**Figure 3:**
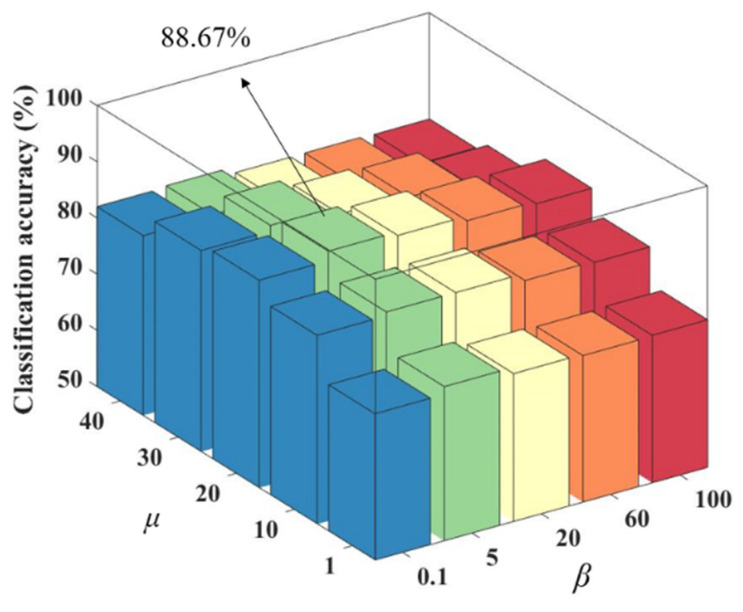
Classification accuracy of different regularization parameter combinations.

The value of *K* is fixed after determining the optimal number of nearest neighbors as 7. The optimal values of these regularization parameters can be searched by varying the values of the group sparsity regularization parameter μ and the adaptive similarity learning regularization parameter *β*. Finally, this regularization parameter combination can help the method get the best classification performance. Figure 3 shows the classification accuracy results for different regularization parameters. As well, we chose different colors in the figure to make the results more intuitive and beautiful.

As seen in Figure 3, among different regularization parameter combinations, μ has a greater impact on the classification accuracy compared to *β*. When μ is held constant, $\beta \left(\mu \right)$ remains relatively stable. When *β* is held constant, $\mu \left(\beta \right)$ shows a trend of increasing and then decreasing classification accuracy as its value increases. The reason for this trend may be that μ affects the sparsity of the matrix ***W***, which determines the number of features. If μ is too small, some features may be ignored, while if it is too large, redundant features may be present, both of which can reduce the classification accuracy. The number of nearest neighbors *K* is set to 7, μ is set to 20 and *β* is set to 5.

### 3.2. Contrast Experiment

In the experiment, nine methods are selected for comparison to validate the effectiveness of the proposed framework. The baseline methods include MKSVM [33], MKSVM with Lasso feature selection performed independently on single modality (Lasso-MKSVM) [35], manifold regularized-based multimodal feature selection (M2TFS) [15], hypergraph-based multimodal feature selection (HMTFS) [16], ASMFS [17], self-expression topological manifolds-based multimodal feature selection (SETMFS) [18], multimodal classification framework based on ordinary features’ latent relation (OLR), unimodal fMRI and unimodal ASL. The first six methods and unimodal fMRI, along with the proposed framework, depend on SHMR-based brain functional networks to obtain hypergraph feature matrices. In contrast, OLR depends on a lower-order brain functional network constructed solely based on the *Pearson* correlation to obtain ordinary feature matrices. All the above-mentioned, including the proposed framework, are evaluated via ten-fold cross-validation. Furthermore, all methods exploit grid search to obtain the optimal classification performance results. Table 2 shows the specific classification performance, where the best classification results are highlighted in bold.

The ACC, AUC, SPE, and SEN obtained by the proposed method are 88.67 ± 0.08%, 86.20 ± 0.16%, 93.50 ± 0.10%, and 86.00 ± 0.17%, respectively. It can be observed in Table 2 that HLR outperforms the other nine methods in all metrics except for SEN in the ESRDaMCI classification. The classification performance of unimodal fMRI and ASL is completely lower than that of multimodal classification methods when also using MKSVM. It proves that multimodal classification methods combined with different brain imaging modalities can reveal the functions and features of brain networks from different perspectives, and it also proves that joint fMRI and ASL can better improve classification performance. HMTFS demonstrates better classification performance compared to the first five methods, indicating that the hypergraph regularization term can discover high-order relations between features. ASMFS performs better than HMTFS, suggesting that dynamically capturing the intrinsic similarity shared by different modalities can select more informative features for classification. The classification accuracy of SETMFS reached 85.83 ± 0.10%, indicating that adopting a topology manifold to compute the similarity between data points within the feature matrix outperforms the traditional use of Euclidean distance as a similarity measure. This approach fully considers the topological relationships among data points from different modalities, which is conducive to improving classification performance.

Nonetheless, HLR outperformed SETMFS in terms of classification performance, suggesting that solely considering the topological relationships between different modalities is not as effective as mining the latent relation among feature matrices to select more useful feature information. The latent relation matrix, which replaces the original features, is not affected by outliers and noise, providing strong robustness. The constructed similarity matrix contains more manifold information compared to ASMFS and SETMFS. Moreover, HLR performs better classification than OLR. It indicates that the hypergraph features extracted on SHMR-based brain functional networks can enhance classification accuracy compared to ordinary features. The reason for the higher SEN values for OLR than HLR may be that OLR has a higher recognition rate for positive samples. In contrast, HLR has high-order information and is more sensitive to disease information, resulting in a slight decrease in the recognition rate of positive samples.

### 3.3. Discriminative Brain Regions

**Figure 4:**
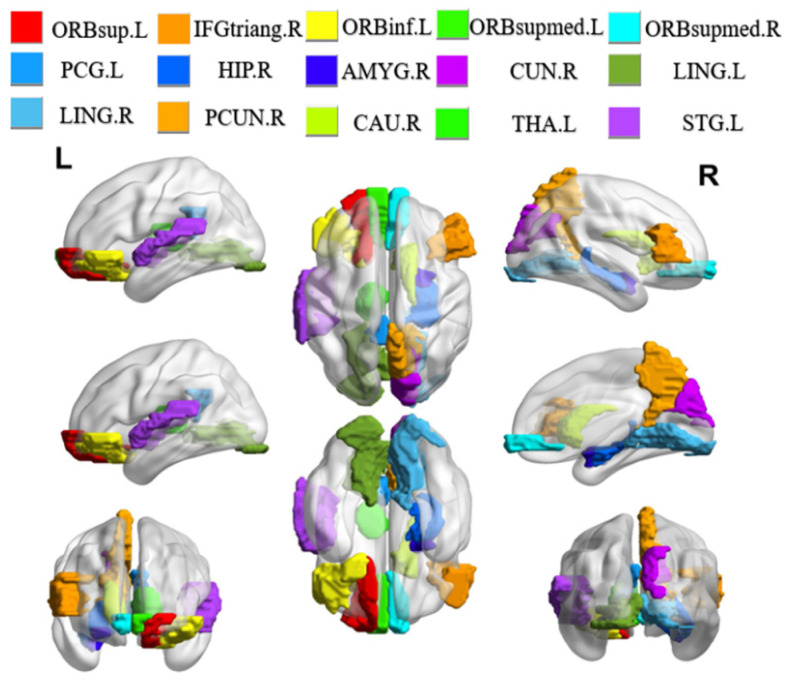
Visualization of discriminative brain regions. L and R represent the left and right directions, respectively.

We conduct experiments to seek the most discriminative brain regions in ESRDaMCI classification after determining the optimal parameter combination through ten-fold cross-validation. The weight matrix ***W*** is analyzed and sorted, and the top fifteen brain regions with the highest weights are selected as the discriminative brain regions. The BrainNet Viewer toolbox is utilized to visually showcase these regions, and the results are shown in Figure 4. This visualization allows for a more straightforward presentation of the spatial locations and relations among the selected discriminative brain regions.

It is found that the selected discriminative brain regions are predominantly located in the frontal lobe through the analysis of the results. These regions include the left orbital part of the superior frontal gyrus (ORBsup.L), the right triangular part of the inferior frontal gyrus (IFGtriang.R), and the left orbital part of the inferior frontal gyrus (ORBinf.L), among others. The frontal lobe plays a significant role in brain memory, judgment, and abstract thinking [36]. As a result, these selected brain regions indicate that individuals with ESRDaMCI have experienced changes in functions such as memory and judgment compared to healthy individuals. In addition, the chosen discriminative brain regions include the right hippocampus (HIP.R), right caudate nucleus (CAU.R), left lingual gyrus (LING.L), and right lingual gyrus (LING.R), which play essential roles in human memory and spatial localization abilities [37]. The left thalamus (THA.L) plays a crucial role in sensory reception functions [38], while the right precuneus (PCUN.R) and right cuneus (CUN.R) are associated with various high-order cognitive functions [39]. These findings are consistent with previous pathological studies and reports on neuroimaging biomarkers of ESRDaMCI [40,41]. They validate our results from the perspective of overall brain function, revealing the discriminative brain regions underlying ESRDaMCI and providing clinical significance for diagnostic purposes.

### 3.4. Data Visualization and Analysis

**Figure 5:**
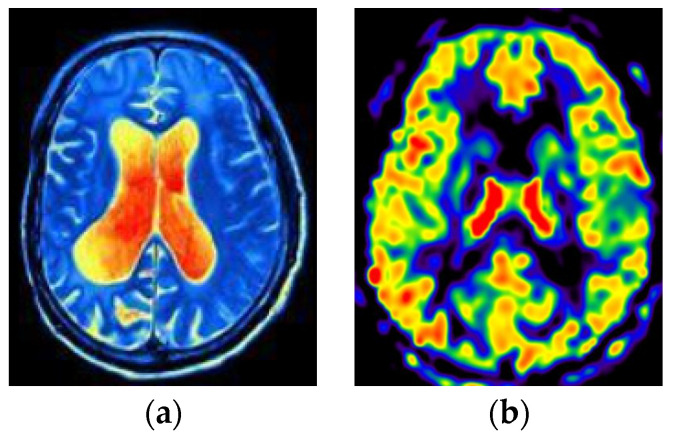
Visualization of fMRI and ASL data. (**a**) fMRI (**b**) ASL.

This section visualizes the ASL and fMRI data images, as shown in Figure 5.

In this study, fMRI is processed as the first modal data and ASL as the second modal data. The combination of these two modal data can better collect feature information inspired by [8,9]. First, different modes provide different types of information. fMRI shows the temporal and spatial distribution of brain activity, ASL provides information on blood flow in brain regions, and both modes provide more comprehensive information. Next, fMRI can be affected by noise, while ASL provides blood flow information that reduces potential errors. Finally, the combination of fMRI and ASL can better observe the changes in brain regions, which is convenient for finding the discernible brain regions of ESRDaMCI.

## 4. Discussion

In recent years, there has been increasing attention from researchers on MCI-related diseases [42,43,44,45], including ESRDaMCI, which has gained significant importance. However, the underlying pathological mechanisms of the disease remain unclear, making the classification and identification of related diseases a challenge. Multimodal learning has been introduced in the field of medical imaging, allowing researchers to gain a more comprehensive understanding of complementary information from different modalities, thereby aiding in the diagnosis and identification of ESRDaMCI. High-dimensional features have a significant impact on the final performance of the models in multimodal classification. Existing multimodal disease classification methods mostly focus on improving feature selection algorithms, but these methods have varying degrees of limitations and issues. HMTFS proposed by Shao et al. [16] considers the high-order relations between different modalities and gets good classification performance. It neglects whether the feature matrix accurately reflects the high-order relations between brain regions, thereby limiting its ability to discover discriminative brain regions for imaging diseases. M2TFS, proposed by Jie et al. [15], constructs a fixed similarity matrix, failing to reveal the underlying data structure. ASMFS, proposed by Shi et al. [17], updates the similarity matrix in real-time, but it does not consider outliers and noise in the features. SETMFS proposed by Song et al. [18] only focuses on the topological relationships between different modalities without considering the latent relation among features within each modality and the handling of noise. They also neglect the quantity and quality of useful information contained in the original feature matrix.

These methods fail to gather more high-order prior information on brain regions and exploit the latent relation between feature matrices. To address these issues, HLR constructs SHMR-based brain functional networks with hypergraph structure information and obtains the hypergraph feature matrix with GT when constructing the modality feature matrix. This ensures that the high-order information between brain regions is not overlooked and allows for better capturing of important features in the data [46]. Furthermore, the LRMFS is obtained using LRAS on the basis of multi-task feature selection. This method generates more accurate similarity matrices, ensuring clear features while removing noise from the feature matrix. In particular, previous similarity matrices are constructed from the original feature matrix, ignoring the latent feature relation and noise. Whereas in the real world, the noise, outliers, and special values contained in the data affect the quality of the similarity matrix. If the original feature matrix is robustly decomposed, it constructs a similarity matrix unaffected by noise and has high information content. The feature matrix in the HLR is robustly structured to mine the feature information between the features, control the noise effects and improve the classification performance.

It is worth noting that this study involves the same original dataset as Song et al., yet the classification performance of HLR surpasses that of SETMFS. It indicates that merely changing the method of computing similarity to capture the topological relationship between feature matrices does not suffice for better extracting latent relation between them. HLR uses latent relation matrices instead of feature matrices to construct the similarity matrix, thereby reducing the impact of noise and outliers on classification performance. Then the feature matrix of SETMFS focuses only on the relational information of pairs of brain regions, and HLR retains the high-order relational information of multiple brain regions, identifying more accurate discriminative brain regions. Overall, HLR changes the feature matrix attributes so that the feature matrix has high-order information. It also proposes a new LRMFS method that changes the construction of the similarity matrix to mine the potential relationships between the data and improve the robustness of the model.

In addition, HLR is a multimodal classification framework based on the combination of ASL and fMRI. Studies have shown that these two modalities provide a more comprehensive understanding of brain information [8,9]. On the one hand, ASL measures CBF to reflect brain metabolism, while fMRI detects changes in blood oxygen levels to reflect functional brain activity. Combining them allows for a more comprehensive representation of neural activity. Additionally, ASL provides temporal data of neural activity through CBF values, while fMRI provides high spatial resolution brain activity images, enabling the correspondence between time and space. On the other hand, the blood flow data in ASL are closely related to brain metabolism and neural activity, while the blood oxygen level changes in fMRI reflect the demand for neural activity. Therefore, combining these two modalities can provide more accurate and comprehensive physiological interpretability.

The experimental results demonstrate that HLR outperforms the comparison methods regarding classification performance and obtains significant results in identifying subtle changes in ESRDaMCI brain regions. It has some significance for the clinical diagnosis of ESRDaMCI. Nevertheless, HLR still has certain limitations and requires further improvement. Traditional Euclidean distance calculation in constructing the similarity matrix may not be suitable for capturing complex network topology structures [47]. In the upcoming work, we will integrate topological manifold terms [18] to compute the topological relationship matrix between features, thereby obtaining a more accurate similarity matrix. HLR involves many parameters and requires multiple parameters tuning to get the optimal model, and the process is complex, requiring further model refinement. Moreover, deep learning is also developing rapidly in the medical field [48,49,50,51], and HLR can adopt graph convolutional networks to construct more appropriate dynamic hypergraphs that retain more a priori information about the brain. Finally, HLR shows good performance on the binary classification problem of ESRDaMCI, but it can be further extended to the multiclassification problem, which is able to recognize and classify ESRDaMCI with different degrees of refinement.

In summary, the HLR-based multimodal classification framework has the following advantages and contributions. (a) The feature matric retains the priori information between multiple brain regions rather than between pairs of brain regions. It better reflects the changes in ESRDaMCI brain regions and contributes to obtaining more accurate discriminative brain regions. (b) A new feature selection method of LRMFS is proposed, which utilizes the original features to construct coefficient matrices with good noise immunity. It adapts adaptive learning and latent relation learning to combine in a multi-task feature selection model to explore the latent relation between features and improve the robustness of the model. (c) The current status of the development of two types of medical images, ASL and fMRI, is examined, and the advantages of combining the two modalities, ASL and fMRI, are explored. (d) The discriminative brain regions of ESRDaMCI are identified by the selected features. It can provide a research basis for the prevention and treatment of ESRDaMCI by determining the subtle changes in these brain regions in medical clinical diagnosis.

## 5. Conclusions

We propose a HLR-based multimodal classification framework and apply it to the identification of ESRDaMCI disease. The HLR, unlike previous studies, achieves joint learning of high-order information in hypergraphs and latent relation feature selection. This framework constructs a hypergraph feature matrix by the SHMR-based brain functional network, providing more high-order prior information about brain regions and identifying discriminative brain regions for ESRDaMCI. It better reflects the pathogenesis of ESRDaMCI through these discriminative brain regions. Moreover, HLR adopts the latent relation in the feature matrix to construct a new feature coefficient matrix, which reduces the impact of noise and enhances robustness. Subsequently, it constructs a similarity matrix that yields greater information and more discriminative features through adaptive learning, thereby improving the classification performance for ESRDaMCI. In clinical diagnosis, it can assist patients in receiving timely treatment, reduce the likelihood of MCI converting to AD, and provide important imaging markers for ESRDaMCI. Nevertheless, our study has many limitations. For example, we construct the similarity matrix according to traditional Euclidean. It tends to neglect the topological relationship between the feature matrices of different modalities. In fact, the framework involves many parameters, and the model optimization process is complicated. Deep learning, especially deep neural networks, will also be applied in recognizing ESRDaMCI to further improve classification performance.

## Figures and Tables

**Table 1:** Demographic information of subjects.

	Gender (Male/Female)	Age $\left(\bar{x}\pm s\right)$	Education Years $\left(\bar{x}\pm s\right)$	MoCA Scores $\left(\bar{x}\pm s\right)$
ESRD group	24/20	49.25 ± 11.15	9.58 ± 2.72	23.87 ± 4.51
NC group	13/31	46.25 ± 11.39	9.65 ± 2.59	26.63 ± 3.93

**Table 2:** Classification performance of different methods.

Method	ACC (%)	AUC (%)	SPE (%)	SEN (%)
fMRI	61.04 ± 0.14	59.76 ± 0.18	56.75 ± 0.23	57.35 ± 0.21
ASL	63.18 ± 0.15	67.84 ± 0.18	51.35 ± 0.23	75.00 ± 0.22
MKSVM [33]	73.93 ± 0.13	63.75 ± 0.24	62.07 ± 0.36	82.65 ± 0.14
Lasso-MKSVM [35]	76.17 ± 0.12	76.60 ± 0.17	67.95 ± 0.23	84.55 ± 0.16
M2TFS [15]	67.90 ± 0.11	56.15 ± 0.28	59.78 ± 0.35	85.40 ± 0.18
HMTFS [16]	81.14 ± 0.16	81.63 ± 0.18	77.00 ± 0.22	79.00 ± 0.18
ASMFS [17]	85.08 ± 0.16	82.28 ± 0.21	78.00 ± 0.28	88.50 ± 0.12
SETMFS [18]	85.83 ± 0.10	83.47 ± 0.24	86.31 ± 0.19	84.97 ± 0.23
OLR	84.42 ± 0.09	80.55 ± 0.13	83.50 ± 0.17	**90.00 ± 0.17**
HLR	**88.67 ± 0.08**	**86.20 ± 0.16**	**93.50 ± 0.10**	86.00 ± 0.17

## Data Availability

The data presented in this study are available on request from the corresponding author. The data are not publicly available due to privacy and ethical.
